# Grading of lung adenocarcinomas with simultaneous segmentation by artificial intelligence (GLASS-AI)

**DOI:** 10.1038/s41698-023-00419-3

**Published:** 2023-07-18

**Authors:** John H. Lockhart, Hayley D. Ackerman, Kyubum Lee, Mahmoud Abdalah, Andrew John Davis, Nicole Hackel, Theresa A. Boyle, James Saller, Aysenur Keske, Kay Hänggi, Brian Ruffell, Olya Stringfield, W. Douglas Cress, Aik Choon Tan, Elsa R. Flores

**Affiliations:** 1 Departments of Molecular Oncology, H. Lee Moffitt Cancer Center, Tampa, 33612 FL USA; 2 Cancer Biology and Evolution Program, H. Lee Moffitt Cancer Center, Tampa, 33612 FL USA; 3 Biostatistics and Bioinformatics, H. Lee Moffitt Cancer Center, Tampa, 33612 FL USA; 4 Quantitative Imaging Core, H. Lee Moffitt Cancer Center, Tampa, 33612 FL USA; 5 Anatomic Pathology, H. Lee Moffitt Cancer Center, Tampa, 33612 FL USA; 6 Immunology, H. Lee Moffitt Cancer Center, Tampa, FL 33612 USA

**Keywords:** Non-small-cell lung cancer, Cancer models, Tumour heterogeneity

## Abstract

Preclinical genetically engineered mouse models (GEMMs) of lung adenocarcinoma are invaluable for investigating molecular drivers of tumor formation, progression, and therapeutic resistance. However, histological analysis of these GEMMs requires significant time and training to ensure accuracy and consistency. To achieve a more objective and standardized analysis, we used machine learning to create GLASS-AI, a histological image analysis tool that the broader cancer research community can utilize to grade, segment, and analyze tumors in preclinical models of lung adenocarcinoma. GLASS-AI demonstrates strong agreement with expert human raters while uncovering a significant degree of unreported intratumor heterogeneity. Integrating immunohistochemical staining with high-resolution grade analysis by GLASS-AI identified dysregulation of Mapk/Erk signaling in high-grade lung adenocarcinomas and locally advanced tumor regions. Our work demonstrates the benefit of employing GLASS-AI in preclinical lung adenocarcinoma models and the power of integrating machine learning and molecular biology techniques for studying the molecular pathways that underlie cancer progression.

## Introduction

The approval of whole slide scanners for use in clinical pathology by the U.S. Food and Drug Administration (FDA) in 2017 led to the rapid proliferation of digital pathology images in both healthcare and pre-clinical settings. Not only have whole slide images (WSIs) increased the efficiency of pathologists’ workflow, but their digitization also enables collaboration among geographically distant groups. Furthermore, advances in computer vision and image processing have given rise to several applications that can assist in the histopathological analysis of WSIs, particularly in the field of oncology. These applications often utilize pre-trained convolutional neural networks (CNNs) to perform or assist with time-consuming tasks, such as nuclei segmentation^1,2^, histological staining analysis^3^, and tumor segmentation^4–6^. Similar machine-learning approaches have been developed for more nuanced analyses, including quantifying tumor-associated or tumor-infiltrating immune cells^7–9^, microsatellite instability^10^, and prediction of patient mutational status from WSIs^11,12^. Machine learning models trained to classify tumors into diagnostically distinct grades using existing systems, such as the Gleason score for prostate cancer^13–15^, have also been reported. In many of these studies, the accuracy of the machine-learning model has been measured in terms of agreement with expert human raters on a sample-by-sample basis. While a suitable performance measure, this comparison level fails to capture much of the information uncovered by the high-resolution analysis these algorithms perform.

In addition, the development of these machine-learning models has been focused almost exclusively on analyzing human samples. These efforts benefit tremendously from publicly available data sets from human patients, like those stored in The Cancer Genome Atlas (TCGA)’s collection of WSIs and associated molecular data^16^. However, the intense focus on clinical applications has provided few machine-learning models useful for translational and basic research projects that rely on pre-clinical animal models.

Machine learning applications in pre-clinical research present an excellent opportunity to enhance and accelerate analyses of the experimental data produced from these efforts. Several genetically engineered mouse models of lung adenocarcinoma (LUAD) have been reported, of which the *Kras*^LSL-G12D/+^ model is the most widely used^17^. This well-studied mouse model is a valuable baseline for studying other mutations commonly found in LUAD, such as *Trp53*^R172H^, separately or in conjunction with the activating *Kras*^*G12D*^ mutation. Unlike human patients, these mouse models often develop over 100 primary tumors of varying histological grades throughout the lungs, which makes thoroughly analyzing these valuable specimens extremely time-consuming, even for experienced cancer researchers or clinicians.

Here, we report GLASS-AI (Grading of Lung Adenocarcinoma with Simultaneous Segmentation by Artificial Intelligence), a machine learning pipeline for the analysis of mouse models of lung adenocarcinoma that provides a rapid and reproducible means of analyzing tumor grade from WSIs. Analysis of several mouse models of LUAD revealed a high degree of accuracy comparable to expert human raters. Furthermore, the high-resolution analysis performed by GLASS-AI revealed extensive intratumor heterogeneity that was not reported by the human raters. Alignment of these heterogeneous tumor regions with adjacent immunostained sections showed a strong correlation between tumor grade and aberrant Mapk/Erk signaling that differed between *Kras*;^G12D/+^ *Rosa*^mG/mG^ (K), *TAp73*;^∆td/∆td^ *Kras*^G12D/+^ (TK), and *Kras*;^G12D/+^ *Trp53*;^R172H/+^ *Rosa*^mG/mG^ (KP-R172H) mouse models. The GLASS-AI pipeline empowers pre-clinical research by rapid, reproducible analysis of LUAD without requiring extensive training of human raters.

## Results

### Training of machine learning model

Developing an accurate machine learning model requires a large amount of high-quality training data. To construct our training dataset, we collected WSIs from *Kras*;^G12D/+^ *Rosa*^mG/mG^ (K) (*n* = 4), *TAp73*;^∆td/∆td^ *Kras*^G12D/+^ (TK) (*n* = 15), and *Kras*;^G12D/+^ *Trp53*^*∆/∆*^ (*n* = 14) mice 30 weeks after LUAD initiation. Kras^G12D^ mice and other mice with additional deletions of tumor suppressor proteins (i.e., Trp53) or protein isoforms (i.e., the TA isoform of Trp73) were used to ensure that low, medium, and high-grade tumors present in our training data. The 33 WSIs were divided among three expert human raters, ensuring each rater had at least one WSI from each mouse genotype and randomly assigning the remainder. The raters then segmented and graded tumors throughout the WSIs using the mouse LUAD grading scale previously reported^18,19^ (Supplementary Fig. 1).

**Fig. 1:**
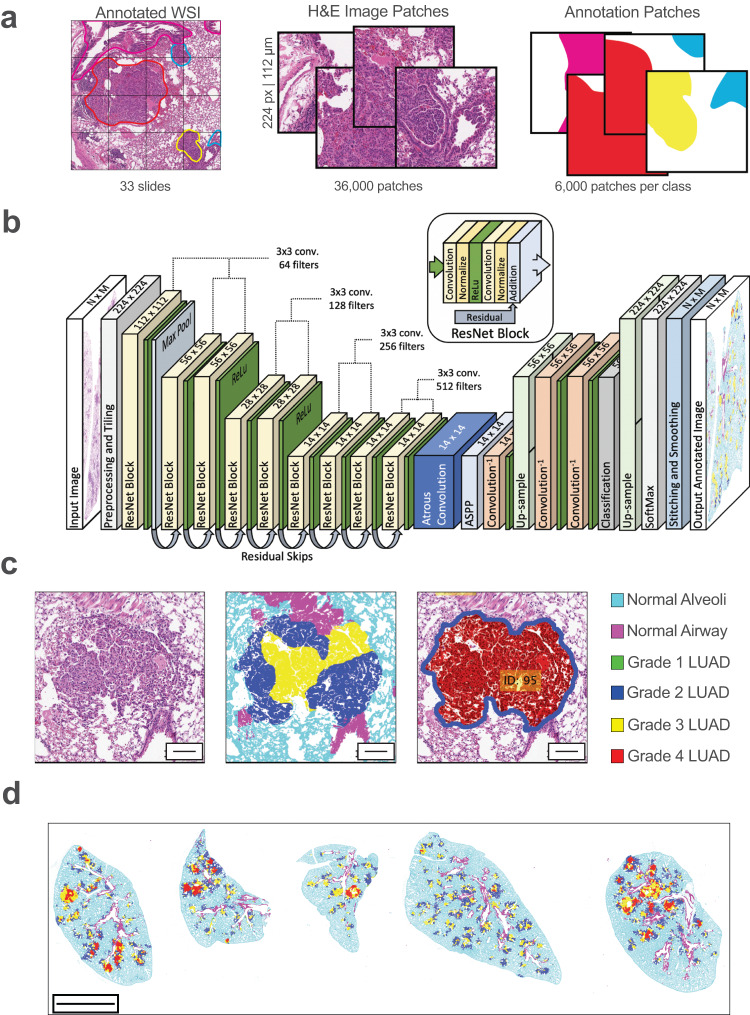
Supervised training of GLASS-AI machine learning model. **a** The GLASS-AI training dataset was generated from 33 whole slide images from three mouse models of lung adenocarcinoma (LUAD) analyzed by human graders. WSIs were divided into 224 × 224 pixel (112 × 112 µm) patches, and 6000 patches for each of the six classes were used to train our CNN. **b** Diagram of GLASS-AI network architecture. **c** Example region from input H&E WSI (left), tumor grading map (middle), and tumor segmentation map (right) produced by GLASS-AI. **d** Example of complete grading map produced by GLASS-AI. Scale bars represent 100 µm (**c**) or 1 mm (**d**).

The annotated WSIs were then used to build a training library of ~6000 patches for each of the six target classes that were then split 60/20/20 for model training, validation, and testing (Fig. 1a). Data augmentation was used to ensure that the total area of each of the target classes was equally represented within each of the training, validation, and testing datasets (Supplementary Fig. 2). Our machine learning model was based on ResNet18^20^ with a rectified linear unit (ReLU)-only pre-activation (Fig. 1b). Transposed convolutional layers were included after the ResNet layers to output graphical maps of tumor grading calls (Fig. 1c, middle) and segmented tumors (Fig. 1c, right), in addition to tabulation of areas of each grade within each segmented tumor and the whole image. WSIs can be input directly into GLASS-AI to quickly identify and grade the tumors throughout a lung cross-section (Fig. 1d).

After training, GLASS-AI achieved an accuracy of 88% on the patches in the final testing data set. However, the image patches used in this assessment only partially capture segmentation and classification accuracy due to their small size and disconnected nature. To test the accuracy of GLASS-AI on complete specimens, we collected a subset of 10 WSIs (5 Kras;^G12D/+^ Rosa^mG/mG^ and 5 TAp73;^∆td/∆td^ Kras^G12D/+^) from a new cohort of mouse models of LUAD that were collected after GLASS-AI had been trained. These 10 WSIs contained 1958 tumors manually segmented and graded by a fourth human rater who did not contribute to the annotation of the model training dataset.

**Fig. 2:**
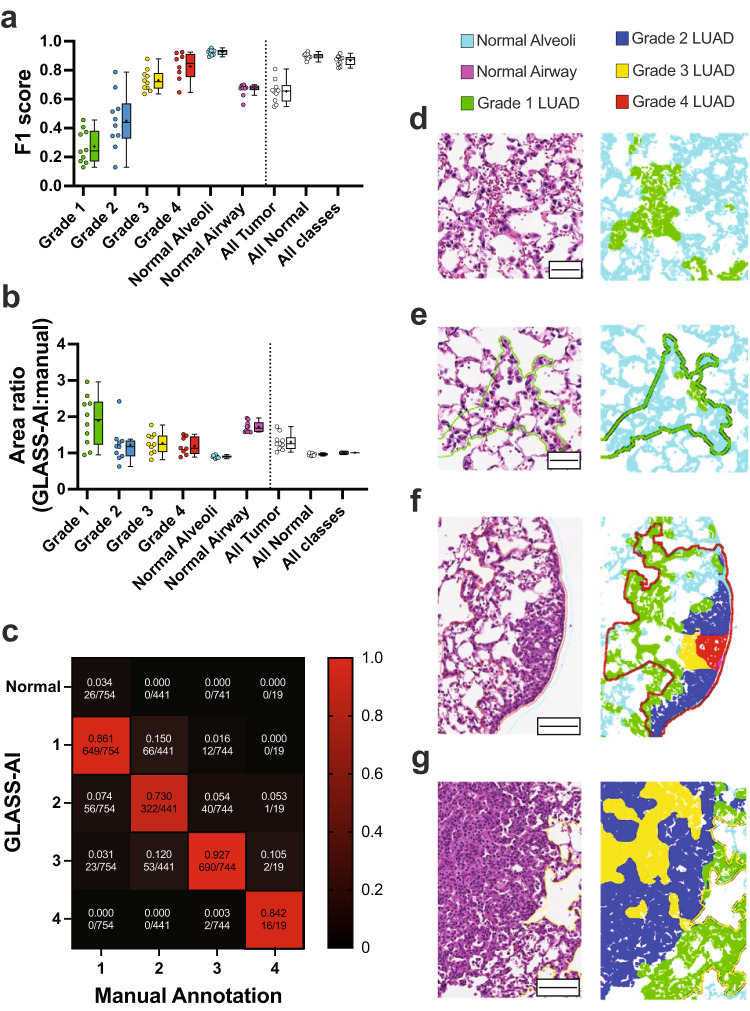
Comparison of manual annotation and GLASS-AI on WSIs. **a**–**g** An additional set of 10 WSIs from Kras;^G12D/+^ Rosa^mG/mG^ (*n* = 5) and TAp73;^∆td/∆td^ Kras^G12D/+^ (*n* = 5) mice were analyzed by an expert human rater who did not contribute to the generation of the training image library for GLASS-AI. After assigning overall grades to the tumors annotated by GLASS-AI based on the highest tumor grade present that comprises ≥ 10% of the tumor area, the performance of GLASS-AI was measured using F1-score (**a**) and annotated area ratios (**b**) for each class in each slide. Boxplots are presented in Tukey style; a line at the median with IQR, crosses indicating the mean, and whiskers showing the lesser of 1.5× IQR or most extreme values. Points show individual values for each slide (**a**, **b**). In addition, the grading of GLASS-AI within the manually annotated regions was used to compare grading accuracy to the manual grading (**c**). Examples of H&E images and GLASS-AI grading with manual annotations overlays are provided to show Grade 1 LUAD that was found by GLASS-AI and missed by the human rater (**d**), Grade 1 LUAD that was found by the human rater and missed by GLASS-AI (**e**), and tumors with regions of heterogenous grades found by GLASS-AI (**f**, **g**). Scale bars represent 50 µm (**d**, **e**) or 100 µm (**f**, **g**).

After segmentation by GLASS-AI, each tumor in WSIs was assigned an overall grade tumor using the same criteria employed by the human raters;^21^ overall tumor grades were assigned based on the highest tumor grade present that comprised at least 10% of the tumor’s area. GLASS-AI achieved a Micro-averaged F1-score of 0.867 (global precision = 0.864, global recall = 0.869) across the 10 WSIs. Examining the F1-score for each class showed a trend toward higher scores with increasing tumor grade (Fig. 2a). By comparing the ratio of the tumor areas annotated by GLASS-AI and the human rater, we found that GLASS-AI annotated an average of 31% more tumor area. This increase was most pronounced in the Grade 1 tumors (Fig. 2b), which are usually smaller and more difficult to notice than tumors of higher grades. Reviewing the Grade 1 areas identified by GLASS-AI and not the human rater showed that a number of these regions were likely Grade 1 LUAD or atypical adenomatous hyperplasia that was missed by the human rater (Fig. 2d). We also observed a large increase in the amount of normal airway area found by GLASS-AI. Upon inspection, we found that this was due to the misclassification of the smooth muscle cells of the pulmonary arteries, a cell type also surrounding the airways of the lung (Supplementary Fig. 3).

GLASS-AI successfully recognized tumors within 1932 of the 1958 manually segmented tumors within the 10 WSIs (Table 1). All 26 of the tumors missed by GLASS-AI were manually annotated as Grade 1 and were classified as “normal alveoli” (Fig. 2e). In addition to identifying 98.7% of manually annotated tumors, GLASS-AI’s segmentation also covered 90% of the manually annotated tumor area. To directly compare overall tumor grading between GLASS-AI and the human rater, the manually annotated regions were combined with GLASS-AI’s grading to assign the overall tumor grade to each manually segmented tumor. GLASS-AI and the human rater assigned the same grade to 1677 (85.6%) of the annotated tumors resulting in a Cohen’s kappa of 0.782 (95%CI: 0.759–0.806) with a linear weighted kappa of 0.835. We observed that the grading agreement was high across all four tumor grades (Fig. 2c) despite the high degree of intratumor heterogeneity found in LUAD tumors (Fig. 2f, g). GLASS-AI also analyzed all 10 WSIs in 75 min, at ~7.5 min per slide, while the human rater required ~4.5 h per slide. The speed, accuracy, and resolution of GLASS-AI can greatly empower the analysis of mouse models of LUAD.

**Table 1:** Overall tumor grade assignment by human raters and GLASS-AI.

GLASS-AI grade	Manual annotation				
	Grade 1	Grade 2	Grade 3	Grade 4	Total
Normal	26	0	0	0	26
Grade 1	649	66	12	0	727
Grade 2	56	322	40	1	419
Grade 3	23	53	690	2	768
Grade 4	0	0	2	16	18
Total	754	441	744	19	1958

### GLASS-AI analysis of mouse models of lung adenocarcinoma

**Fig. 3:**
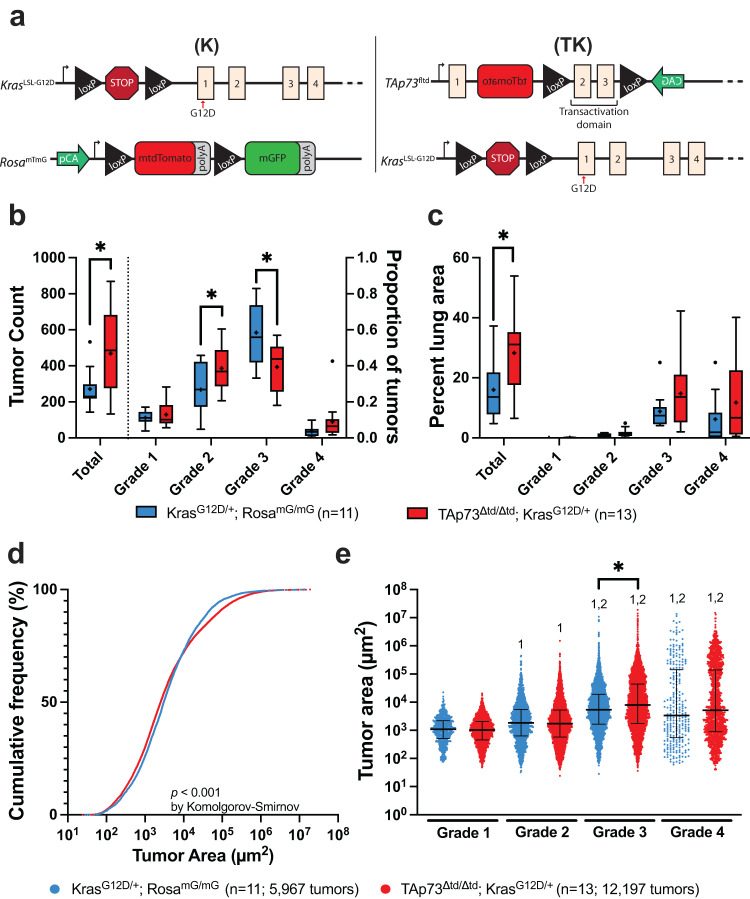
GLASS-AI analysis of mouse models of LUAD. **a**–**e** A cohort of Kras;^G12D/+^ Rosa^mG/mG^ (K) (*n* = 11) and TAp73;^∆td/∆td^ Kras^G12D/+^ (TK) (*n* = 13) mice were analyzed by GLASS-AI (**a**). After tumor segmentation and grading, the number of lung tumors (**b**) and tumor burden (**c**) for each mouse were totaled and analyzed by two-tailed Student’s *t*-test with Holm-Šídák correction for multiple comparisons. Each tumor was assigned a single grade based on the highest grade present that comprised ≥10% of the tumor’s area. Boxplots are presented in Tukey style; a line at the median with IQR, crosses indicating the mean, whiskers showing the lesser of 1.5× IQR or most extreme values, and points indicating outliers > 1.5× IQR from the median (**b**, **c**). The distribution of individual tumor sizes was examined by calculating the cumulative frequency of all grades of tumors in each genotype and was analyzed using a two-tailed Komolgorov-Smirnov test (**d**). Individual tumors were grouped by genotype and grade to perform a more in-depth size distribution analysis and analyzed by one-way ANOVA with two-tailed Tukey’s post-hoc test after log transforming the tumor area. Lines indicate median values, and whiskers show IQR (**e**). *p < 0.05 for the indicated comparison between K and TK mice (**b**, **c**, **e**). Numerals indicate *p < 0.05 between the noted grade within the same genotype (**e**).

The initial test of the GLASS-AI pipeline was carried out on *Kras*;^*G12D/+*^ *Rosa*^*mG/mG*^ (K) and *TAp73*;^∆td/∆td^ *Kras*^*G12D/+*^ (TK) mouse models (Fig. 3a) to assess the utility of GLASS-AI for comparing the experimental TK mouse model of LUAD to an existing, well-characterized K LUAD model. The tumor phenotypes of the K mouse model have been characterized by previous studies^17,19,22^, while the tumor phenotypes in the TK model are unknown. These comparisons were carried out with the entire cohort of 11 K and 13 TK mice that contained the subset of 10 WSIs analyzed in Fig. 2. To directly assess changes in tumor progression associated with the loss of TAp73, the mice used for this study were collected 30 weeks after initiation of LUAD by intratracheal instillation with adenovirus expressing *Cre* recombinase under the control of a CMV promoter.

After analyzing the cohort of 11 K and 13 TK mice with GLASS-AI, we found that the average number of tumors in TK mice was 73% higher than in K mice (469 [95%CI = 322–470] vs. 271 [95%CI = 199–344], *p* = 0.021, Student’s t-test statistic t = 2.48, df=22, Cohen’s *d* = 1.049) (Fig. 3b, left axis). GLASS-AI found that the majority of tumors in the K mice were Grade 3, with only a few Grade 1 or Grade 4 tumors. However, the TK mice exhibited a significant decrease in the average proportion of tumors rated as Grade 3 compared to K mice (39.4% [95%CI = 31.3–47.5] vs. 58.5% [95%CI = 47.2–69.7, *p.adj* = 0.0206, Student’s *t*-test statistic *t* = 3.10, df=22, Cohen’s *d* = 1.258). This shift away from Grade 3 tumors was accompanied by an increase in the average proportion of Grade 2 (38.7% [95%CI = 31.2–46.1] vs. 27% [95%CI = 17.6–36.0], *p.adj* = 0.1028, Student’s *t*-test statistic *t* = 2.24, df = 22, Cohen’s *d* = 0.913) and Grade 4 tumors (9.0% [95%CI = 2.4–15.6] vs. 3.5% [95%CI = 1.7–5.3], *p.adj* = 0.2274, Student’s *t*-test statistic *t* = 1.61, df = 22, Cohen’s *d* = 0.687) that did not reach statistical significance (Fig. 3b, right axis).

In addition, the total proportion of lung area occupied by tumors annotated by GLASS-AI in TK mice was significantly higher than in K mice (28.3% [95%CI = 20.5–36.1] vs. 16.0% [95%CI = 9.6–22.4], *p* = 0.0159, Student’s *t*-test statistic *t* = 2.61, df = 22, Cohen’s *d* = 1.084). When examined in more detail, TK mice exhibited an increase in the percentage of lung area filled by tumors of each grade of LUAD, particularly Grade 3 (14.8% [95%CI = 7.5–22.1] vs. 8.8% [95%CI = 4.8–13.0], *p.adj* = 0.3282, Student’s *t*-test statistic *t* = 1.48, df = 22, Cohen’s *d* = 0.624) and Grade 4 (11.8% [95%CI = 4.2–19.4] vs. 6.2% [95%CI = 0.8–11.6], *p.adj* = 0.3282, Student’s *t*-test statistic *t* = 1.26, Cohen’s *d* = 0.528) that did not reach statistical significance (Fig. 3c). Interestingly, when we examined the percent of lung area of each tumor grade found by GLASS-AI without assigning overall grades to the tumors, we found that TK mice had a significant expansion of Grade 2 area compared to K mice (11.3% [95%CI = 7.7–15.0] vs. 4.3% [95%CI = 2.7–5.9], *p.adj* = 0.0052, Student’s *t*-test statistic t = 3.41, df=22, Cohen’s *d* = 1.417). This expansion was proportionally larger than the Grade 3 (11.2% [95%CI = 7.2–15.1] vs. 8.5% [95%CI = 4.6–12.4], *p.adj* = 0.1702, Student’s *t*-test statistic *t* = 1.78, df = 22, Cohen’s *d* = 0.726) and Grade 4 expansions (4.9% [95%CI = 1.1–8.6] vs. 2.9% [95%CI = 0.2–5.7], *p.adj* = 0.1702, Student’s *t*-test statistic *t* = 1.69, df = 22, Cohen’s *d* = 0.679), which did not reach statistical significance (Supplementary Fig. 4). Since we did not observe a large increase in the area of Grade 2 tumors, we reasoned the increased Grade 2 area identified by GLASS-AI would be in Grade 3 or Grade 4 tumors. Indeed, we found that the percentage of total Grade 2 area in all Grade 3 tumors increased from 25% in K mice to 43% in TK mice and likewise increased from 17 to 26% in all Grade 4 tumors (Supplementary Fig. 5).

We next examined the distribution of individual tumor sizes to determine if the increased tumor burden observed in TK mice compared to K mice was due solely to the increased tumor number. Interestingly, while the median tumor size of TK mice was found to be significantly smaller than K mice (2542 µm^2^ [95%CI = 2429–2643] vs. 3093 µm^2^ [95%CI = 2889–3240], *p* = 0.0026, Mann–Whitney test statistic *z* = −3.0149, *r* = 0.022) (Supplementary Fig. S6), a closer examination of the cumulative distribution of tumors revealed that TK mice had a broader distribution of tumor sizes with a higher proportion of smaller and larger tumors than K mice (*p* < 0.0001, Komolgorov-Smirnov test statistic D = 0.051) (Fig. 3d). However, the 6-log range of tumor sizes we observed meant that the more numerous small tumors of the TK mice contributed relatively little to the overall tumor burden. Indeed, 50% of the total tumor area was contained in only 30 (0.5%) and 125 (1.0%) tumors from K and TK mice, respectively. Looking at the distribution of sizes of tumors of each grade in the K and TK mice, we observed that tumors of higher grades were significantly larger than lower-grade tumors, as expected. Furthermore, we noted that the median size of Grade 3 tumors from TK mice was also significantly larger than that of K mice (7929 µm^2^ [95%CI = 7183–8744] vs. 5367 µm^2^ [95%CI = 4971–5774], *p.adj* < 0.0001, Dunn’s multiple comparisons test statistic *z* = 5.927, *r* = 0.067) (Fig. 3e). Therefore, we can conclude that the greater tumor burden of TK mice is due to both the higher number of tumors and the expansion of higher-grade tumors from the increased Grade 2 areas within them.

### Uncovering intratumor heterogeneity

**Fig. 4:**
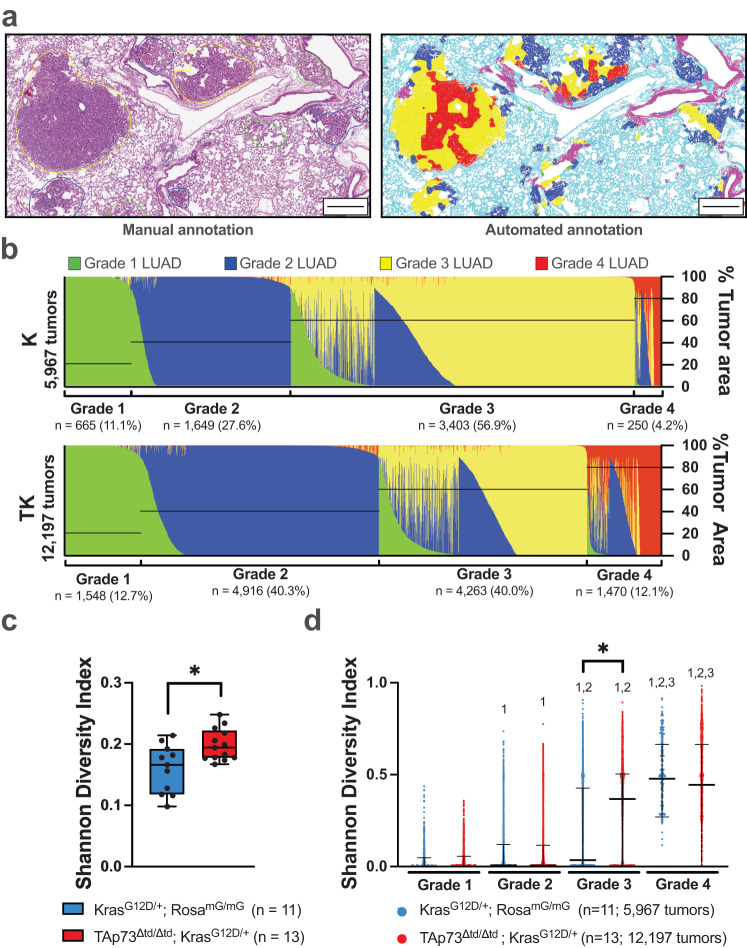
GLASS-AI analysis uncovers intratumor heterogeneity. **a** Representative annotations of tumor grades produced by human raters (left) and GLASS-AI (right). Scale bars represent 200 µm. **b**–**d** The intratumor heterogeneity of individual tumors in the K (*n* = 11) and TK (*n* = 13) mice was analyzed by examining the proportion of each tumor grade within a tumor. Each bar represents a single tumor with the proportion of each grade of LUAD within that tumor shown by the various colors. The black lines overlayed on the graphs and corresponding brackets under the graphs indicate the overall tumor grade assigned to each tumor based on the highest tumor grade present that comprises ≥10% of the tumor’s total area (**b**). The average Shannon Diversity Index (SDI) of each animal’s tumors was calculated and compared by a two-tailed Student’s *t*-test. Boxplots are presented in Tukey style; a line at the median with IQR, crosses indicating the mean, whiskers showing the lesser of 1.5× IQR or most extreme values, and individual values represented by points (**c**). Individual tumors were grouped by genotype and grade to perform a more in-depth analysis of the distribution of intratumor heterogeneity in these mouse models and analyzed by Kruskal–Wallis non-parametric test with two-tailed Dunn’s posthoc test due to high skewness and kurtosis of the SDI values (**d**). Lines indicate median values, and whiskers show IQR (**d**). *p < 0.05 for the indicated comparison between K and TK mice (**c**, **d**). Numerals indicate *p < 0.05 between the noted grade within the same genotype (**d**).

It is important to note that the annotations generated by our expert human raters were based on standard criteria for tumor grading, in which a tumor is assigned a single grade based on the highest grade observed that comprises at least 10–20% of the tumor area^21^. However, GLASS-AI gave grades to individual pixels within the image before tumor segmentation, producing a mosaic of grades within a single tumor (Fig. 4a). This information can be used to understand better the effects of genes of interest and drivers of tumor progression in mouse models of LUAD, including the loss of TAp73 in our LUAD mouse models.

By representing each tumor as a stacked bar divided by the proportion of the tumor area made up of each grade of LUAD, we could visualize the overall distribution of intratumor heterogeneity in the LUAD mouse models (Fig. 4b). From these graphs, we identified patterns in tumor composition, such as the relatively small proportion of Grade 1 area found in Grade 4 tumors or the presence of Grade 2 area in tumors of a higher grade. The shift from predominantly Grade 3 tumors in K mice to other tumor grades, namely Grade 2 and Grade 4, in TK mice was also evident from these graphs (Fig. 4b).

While informative, these visual representations of tumor heterogeneity can provide only a qualitative estimation of heterogeneity in our genetically engineered mouse models of LUAD. To overcome this shortcoming, we employed the Shannon Diversity Index (SDI) as a more quantitative estimate of intratumor heterogeneity. SDI estimates the uncertainty in predicting the grade of a given square micron in a tumor given by SDI $=\,-\mathop{\sum }\nolimits_{i=1}^{n}{p}_{i}{\rm{ln}}{p}_{i}$, where *p*_*i*_ is the proportion of the *i*-th of the *n* present grades from Grade 1 to Grade 4. After estimating the mean SDI from each tumor in a mouse, we found that the TK mice had a higher overall SDI than K mice (0.20 [95%CI = 0.18–0.21] vs. 0.16 [95%CI = 0.13–0.19], *p* = 0.0079, Student’s t-test statistic t = 2.922, df = 22, Cohen’s *d* = 1.511) (Fig. 4c). We also compared the individual tumors of each grade within the K and TK mice and found a trend of increasing heterogeneity with increasing tumor grade in both genotypes (*p.adj* < 0.001 for all comparisons) (Fig. 4d, numerals). This trend is expected due to our overall tumor grade assignment method, which limits low-grade tumors from containing significant amounts of higher-grade regions. However, we observed that the Grade 3 tumors of TK mice had a significantly higher median SDI than K mice (0.37 [95%CI = 0.35–0.38] vs. 0.04 [95%CI = 0.02–0.08], *p.adj* < 0.001, Dunn’s test statistic *z* = 18.75, *r* = 0.2141) (Fig. 4d). These data indicate that the loss of TAp73 increases intratumor heterogeneity, perhaps due to the accumulation of other mutations and defects during tumor progression.

### Aberrant Mek/Erk signaling is associated with grade 4 regions in high-grade tumors

To investigate how the loss of TAp73 contributes to tumor progression and to correlate tumor grading by GLASS-AI with molecular indicators of progression, we performed immunohistochemistry (IHC) for phosphorylated Mek (p-Mek) and phosphorylated Mapk/Erk (p-Erk) on mouse lung sections with adjacent H&E sections graded by GLASS-AI. These downstream effectors of Ras signaling have been previously reported to stain subsets of mouse LUAD tumors and be largely absent in adjacent normal tissue^18^. To facilitate comparisons to these studies, we also analyzed a Kras;^G12D/+^ Trp53;^R172H/+^ Rosa^mG/mG^ (KP-R172H, *n* = 3) mouse model (Supplementary Fig. 7) in addition to the Kras;^G12D/+^ Rosa^mG/mG^ (K, *n* = 3) and TAp73;^∆td/∆td^ Kras^*G12D*/+^ (TK, *n* = 5) mice.

We performed global and local registration on each p-MEK and p-ERK IHC WSI using the H&E-stained WSI as a reference (Supplementary Fig. 8). After image registration, individual cells within each IHC image were segmented and categorized as positive or negative. Using the adjusted coordinates of the registered images, the cells identified in the p-MEK and p-ERK IHC WSIs were then projected back to the tumor grading maps that GLASS-AI produced from the H&E-stained WSI, assigned to the corresponding GLASS-AI output class, and associated with individual tumors in which they were contained. Tumors less than 2000 sq. microns in area or containing fewer than 50 total cells identified from the IHC-stained slide were excluded from the downstream analysis to minimize artifacts from imperfect image registration and cell segmentation.

**Fig. 5:**
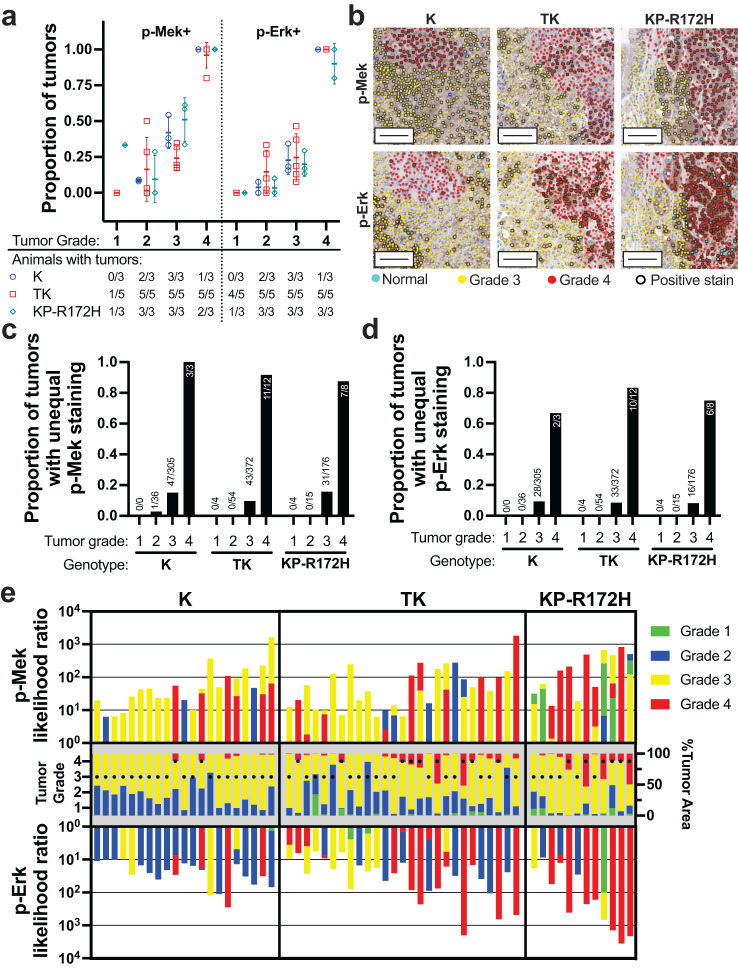
GLASS-AI identifies altered Mek/Erk signaling in high-grade tumor regions. **a** The proportion of LUAD tumors of each grade that were positively stained for p-Mek or p-Erk was determined for each of the K, TK, and KP-R172H LUAD mouse models. Data represent mean ± SD. **b** Overlays of IHC stain analysis and tumor grading from GLASS-AI were generated after registration to adjacent H&E slides. Original magnification ×20, scale bars represent 100 µm. **c**, **d** Positively stained tumors were tested for uneven distribution of p-Mek (**c**) or p-Erk staining (**d**) using a likelihood-ratio G-test. Significance was determined by *p* < 0.01 versus a Chi-squared distribution. **e** Localization of p-Mek and p-Erk enrichment was evaluated using individual likelihood ratios for each grade region within individual tumors that displayed uneven staining distribution of both p-Mek and p-Erk in panels (**c**, **d**). Individual tumors are matched vertically across the three subgraphs. Colors represent the likelihood ratio of each IHC stain within the region of the indicated grade superimposed together (top and bottom) or the percentage of the tumors’ area comprised of each tumor grade (middle). Black dots indicate the overall tumor grade based on the highest tumor grade present that comprises ≥10% of the tumor’s total area (middle).

In all three mouse models, both p-Mek and p-Erk positivity increased with overall tumor grade, and nearly 100% of Grade 4 tumors were positively stained for both markers (Fig. 5a). p-Mek exhibited a broader staining distribution than p-Erk, which primarily appeared in small regions of tumors and occasionally throughout the entire tumor (Fig. 5b). The predominantly focal staining of p-Erk has also been previously reported to occur in high-grade tumors of *Kras*^G12D/+^ mice and mice with additional mutations or deletions of *Trp53*^18,23^.

The high-resolution tumor grading produced by GLASS-AI allowed us to examine the distribution of p-Mek and p-Erk staining within regions of different grades in a single tumor. Tumors that displayed an uneven distribution of positively stained cells were determined using a likelihood-ratio G-test $G=2{\sum }_{i=1}^{4}{O}_{i}{\rm{ln}}\frac{{O}_{i}}{{E}_{i}}$, where *O*_*i*_ is the observed count of positively stained cells in the *i*-th tumor grade and *E*_*i*_ is the expected count of positively stained cells calculated from the total number of positively stained cells in the tumor multiplied by the proportion of the tumor’s area classified as the *i*-th grade by GLASS-AI. These distributions were compared against a Chi-square distribution to determine significance. The proportion of tumors with significantly unequal distribution of either p-Mek or p-Erk was very small in Grade 3 or lower tumors. However, most Grade 4 tumors of all three mouse models displayed significantly disproportionate staining for both markers (Fig. 5c, d). The large increase in the proportion of Grade 4 tumors identified by the G-test compared to the lower grades may indicate that the development of foci of dysregulated Mek/Erk signaling may drive tumor progression.

Based on these observations, we hypothesized that the enrichment of p-Mek and p-Erk staining in the high-grade LUAD of our mouse models should occur in the highest-grade regions of these tumors. By examining the likelihood ratios of the individual grade regions in each tumor, we found that K mice displayed a striking discordance in the enrichment of p-Mek and p-Erk staining, with p-Mek being enriched in higher grade regions in tumors and p-Erk enriching in lower-grade regions of the same tumor (Fig. 5e). In contrast, tumors from TK and KP-R172H mice exhibited a more consistent enrichment of p-Mek and p-Erk staining in the higher-grade regions. We noted that Grade 4 regions exhibited the strongest enrichment for p-Erk even in tumors with a small proportion of Grade 4 area (Fig. 5e, bottom).

## Discussion

Applying machine learning models to digitized WSIs will likely revolutionize how these data are analyzed. Computer vision can assist clinicians by providing rapid screening of images, and the higher resolution analysis performed by machine learning models can uncover features that go unnoticed or unreported by human raters. The analysis of preclinical models will also benefit from employing these machine learning models in analysis pipelines by facilitating rapid, reproducible analysis. In this study, we report a purpose-built neural network for grading lung adenocarcinomas in genetically engineered mouse models that provides an unparalleled identification and analysis of tumor grade heterogeneity. We also demonstrate how this pipeline can be integrated with widely used molecular biology techniques to extend our understanding of the drivers of tumor progression and heterogeneity in LUAD. For example, the consistent enrichment of p-Erk in high-grade regions, even within tumors with a lower overall tumor grade, supports our hypothesis that the localized loss of Mek/Erk regulation beyond the activation of the Ras-Raf-Mek-Erk pathway by oncogenic Kras mutation may drive tumor overall progression. The shift in p-Erk enrichment from low-grade regions in K mice to high-grade regions in TK and KP-R172H mice also highlights the role of the p53 family, and TAp73 in particular, as crucial antagonists of Kras-mediated dedifferentiation in LUAD^24^ and tumor heterogeneity.

Tumor heterogeneity has been implicated in the progression of many cancer types, including non-small cell lung cancers^25,26^. Increased intratumor heterogeneity has been linked to decreased overall survival^25,27,28^, poor response to therapy^29^, and even increased metastasis^30^. This heterogeneity is presumed to arise from the clonal evolution of tumor cells within a neoplasm^31,32^. Typically, tumor heterogeneity is estimated using bulk molecular analyses, such as RNAseq or copy number variation. Previous studies have utilized bulk sample analyses correlated with histomorphological features to predict spatial heterogeneity of molecular markers^33,34^. However, recent studies have begun using spatially sensitive techniques^31^ or multi-region sampling^35^. Combining these approaches with high-resolution analysis from machine learning pipelines like GLASS-AI could provide an unprecedented understanding of cancer development, progression to metastasis, and treatment response through information derived from spatial genomics, transcriptomics, and proteomics correlated with tumor phenotype.

The recent development of commercially available spatial transcriptomics platforms is a promising step forward in correlating molecular and histological analyses. Some groups have begun developing machine learning applications utilizing these technologies^36^. However, these platforms focus on fresh-frozen specimens rather than the FFPE samples typically used for histological analyses in both mouse and human LUAD. Further improvement of these technologies to enable the use of FFPE archival tissues would significantly enhance our understanding of the molecular drivers of tumor progression and heterogeneity and allow the prediction of molecular features from routine histological preparations. This ability to accurately predict molecular markers from simple FFPE, H&E-stained histology images could be used to flag specimens for further molecular characterization and even provide increased diagnostic and therapeutic precision to clinics without regular access to these molecular techniques.

## Methods

### Mouse models and husbandry

*Kras*;^*LSL-G12D/+*^ *Rosa*^*mTmG/mTmG*^ (K), *TAp73*;^*flox-tdTomato/flox-tdTomato*^ *Kras*^*LSL-G12D/+*^ (TK), *Kras*;^*LSL-G12D/+*^ *Rosa*;^*mTmG/mTmG*^ *Trp53*^*LSL-R172H/+*^ (KP-R172H), and *Kras*;^*LSL-G12D/+*^ *Trp53*^*flox/flox*^ mice were generated on a C57BL/6 background. Between 8 and 10 weeks of age, mice were intratracheally instilled with 7.5 × 10^7^ PFU of adenovirus containing *Cre* recombinase under the control of a CMV promoter, as previously described^19^. Mice were euthanized 30 weeks after infection, and lungs were collected, fixed overnight in formalin, and embedded in paraffin for further processing. All procedures were approved by the Institutional Animal Care and Use Committee (IACUC) at the University of South Florida.

### Tissue processing

Formalin-fixed paraffin-embedded (FFPE) lung tissue blocks were sectioned at 4-μm thickness by the Tissue Core at Moffitt Cancer Center. Hematoxylin and eosin (H&E)-stained sections were prepared by the Tissue Core immediately after sectioning. Immunostaining of mouse lung sections was performed overnight at 4 °C in humidified chambers with antibodies against p-Mek1/2 (Ser221) (Cell Signaling Technology Cat# 2338, RRID: AB_490903; 1:200) or p-Mapk (Erk1/2) (Thr202/Tyr204) (Cell Signaling Technology Cat# 4370, RRID: AB_2315112; 1:400) in 2.5% normal horse serum. The IHC signal was developed using DAB after conjugation with ImmPRESS HRP Horse anti-rabbit IgG PLUS polymer kit (Vector Laboratories Cat# MP-7801). Nuclei were counterstained by immersing the slides in Gill’s hematoxylin for 1 min (Vector Laboratories Cat# H-3401).

### Image pre-processing

Whole slide images (WSIs) were generated from H&E and immunostained slides using an Aperio ScanScope AT2 Slide Scanner (Leica) at 20x magnification with a resolution of 0.5022 microns/pixel. To improve the consistency of our pipeline on H&E slides with various staining intensities, staining was normalized using the Macenko method^37^. WSIs of immunostained sections were co-registered to adjacent H&E-stained sections by a combination of global and local co-registration in MATLAB. The global co-registration was achieved by first applying a rigid co-registration to the whole slide of IHC and aligning it to the H&E slide. After the initial rigid alignment, the global co-registration was improved by applying an affine transformation to the IHC slide. This affine co-registration step was lightly applied using only a few iterations to avoid undesired deformation. Local co-registration was then performed by manually aligning tumor regions identified by the pipeline in the H&E image to tumor regions in the IHC slide.

### Machine learning model design

GLASS-AI was written in MATLAB 2021a using the Parallel Processing, Deep Learning, Image Processing, and Computer Vision toolboxes. The standalone GLASS-AI applications for Windows and Mac were built using the MATLAB App Designer and MATLAB Compiler. The network architecture of GLASS-AI was based on ResNet18^20^; an 18-layer residual network pre-trained on the ImageNet dataset^38^. An atrous convolution layer and an atrous spatial pyramid pooling layer were added after the final ResNet18 convolutional layer. The atrous layer performs several simultaneous convolutions on the same input using a set of dilated filters. For example, a 3 × 3 filter with a dilation rate of 4 skips that many pixels between each sampled pixel, effectively spanning a region of 11 × 11. Atrous convolution increases context assimilation by applying multiple dilated filters simultaneously to the input matrix and pooling the results in the next layer. The latent features were then expanded back to the dimensions of the input image patch with transposed convolution before classification. Finally, a smoothing step was added after classification to minimize artifacts from image patch edges. A detailed graphical overview of the network architecture of GLASS-AI is provided in Supplementary Data 1.

### Training image library construction

To construct the training dataset, 33 WSIs were acquired from an available cohort of genetically engineered mouse models of LUAD of varying genotypes (*Kras*;^G12D/+^ Rosa^mG/mG^ *n* = 4, *TAp73*;^∆td/∆td^ *Kras*^G12D/+^ *n* = 15, *Kras*;^G12D/+^ *Trp53*^∆/∆^, *n* = 14). The slides were then divided randomly among three expert human raters, with each rater’s set of 11 WSIs containing at least one slide from each mouse model. The raters manually annotated the individual tumors with grades, normal airways, and normal alveoli throughout each WSI. A total of 6,850 tumors were annotated across the 33 WSIs.

The annotated WSIs were divided into corresponding 224 × 224-pixel image and label patches. Patches were then grouped by the annotated class (Normal alveolar, Normal airway, Grade 1 LUAD, Grade 2 LUAD, Grade 3 LUAD, and Grade 4 LUAD) that was most abundant within each patch; however, all the annotations present within the patches were left intact (i.e., a patch that was predominantly Grade 3 could still contain Normal Alveolar and Grade 4 LUAD annotated pixels). An initial set of 6000 patches were randomly selected for each class from the respective patch group and split 60/20/20 for training, validation, and testing of the machine learning model after ensuring that patches from an individual slide were only present within a single split. Because each image patch could contain varying amounts of each target class, the area of each of the six target classes in each library was balanced via data augmentation by shifting, skewing, and/or rotating patches in which the underrepresented class was the most abundant class present (Supplementary Fig. 2). Using MATLAB Deep Learning Toolbox and 2 NVIDIA P2000 GPUs, the model was set to train for 20 epochs using adaptive moment estimation on 128-patch minibatches with an initial learning rate of 0.01.

### Statistical analysis

Data were analyzed using the statistical tests indicated in the figure legends using GraphPad Prism 9 software. Non-parametric tests were used where indicated to compare data with high skewness or kurtosis. *p* < 0.05 was considered statistically significant unless otherwise stated in the figure legends. Comparisons with more than two groups were first analyzed with an omnibus ANOVA (for parametric) or Kruskal–Wallis test (for non-parametric) before proceeding with the posthoc testing indicated in the figure legends. Family-wise error rate corrections were performed for all multiple comparisons using the method indicated in the figure legends. *P*-values for these comparisons are reported after adjustment (*p.adj*). Effect sizes were calculated using Cohen’s $d=\frac{{\bar{x}}_{1}-{\bar{x}}_{2}}{s}$ with pooled variance for parametric tests and standardized effect size $r=\frac{z}{({n}_{1}+{n}_{2})}$ for non-parametric tests.

### Reporting summary

Further information on research design is available in the Nature Research Reporting Summary linked to this article.

## Supplementary information

Supplementary Information

Supplementary Data 1

REPORTING SUMMARY

## Data Availability

The set of image and label patches used to train GLASS-AI have been deposited in Zenodo (10.5281/zenodo.7967749). The other datasets generated during and analyzed in this study are available from the corresponding author upon reasonable request.

## Code availability

The MATLAB source code for the GLASS-AI standalone applications and links to pre-compiled versions of the application for Windows and MacOS are available at https://github.com/jlockhar/GLASS-AI/. A set of H&E-stained images from a mouse lung adenocarcinoma slide are included for demonstration and benchmarking purposes. GLASS-AI was reported in the AIMe registry for artificial intelligence in biomedical research (https://aime.report/cEX3O6).
