# Supplementary material for: Grading of lung adenocarcinomas with simultaneous segmentation by artificial intelligence (GLASS-AI)
Source: NPJ Precis Oncol. 2023 Jul 18;7:68. doi: 10.1038/s41698-023-00419-3 (PMC10354042; doi:10.1038/s41698-023-00419-3)
Supplement: Supplementary file 1 — Supplementary Information [file 41698_2023_419_MOESM1_ESM.pdf]

Supplementary Information

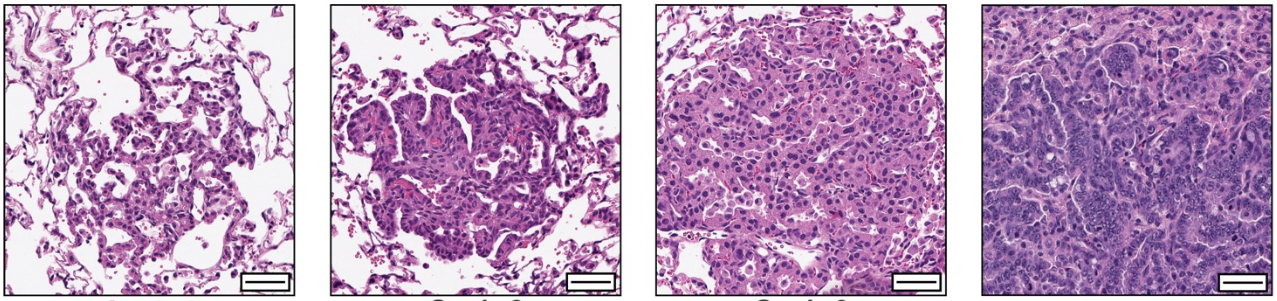

Grade 1
Normal nuclei
Atypical growth pattern

Grade 2
Enlarged nuclei
Visible nucleoli

Grade 3
Deformed nuclei
Prominent nucleoli

Grade 4
Abberant mitosis
Mutlinucleated cells

Supplementary Figure 1: Grades of mouse lung adenocarcinoma. Representative images of Grades 1 – 4 of mouse lung adenocarcinomas based on previously published Lung adenocarcinoma histology grading system for mouse models.

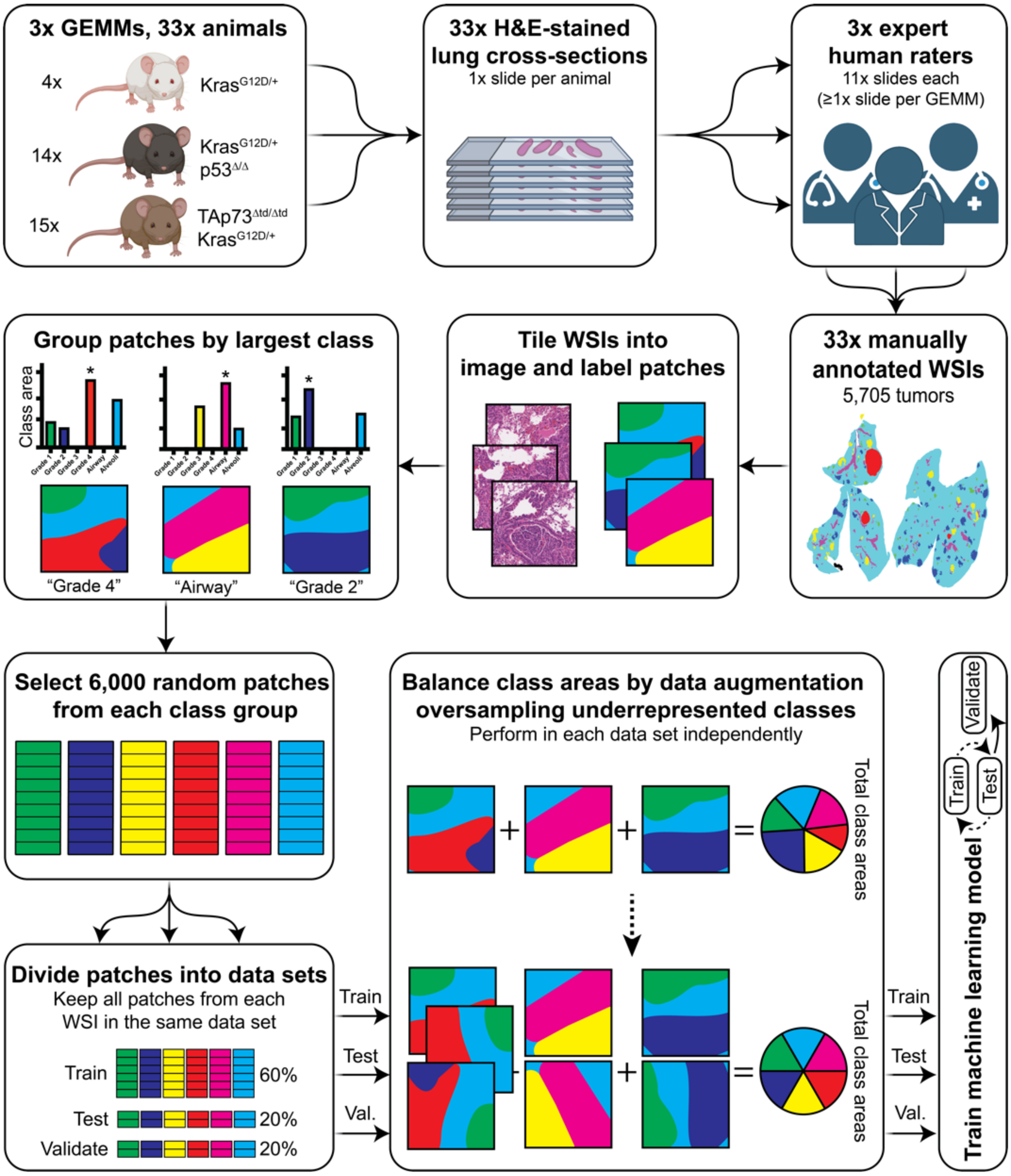

Supplementary Figure 2: GLASS-AI training library construction workflow. Diagram of data sources, divisions, and augmentations employed to construct the image and label patch library for training GLASS-AI. Mouse, microscopy slide, and human rater cartoons were created with BioRender.com.

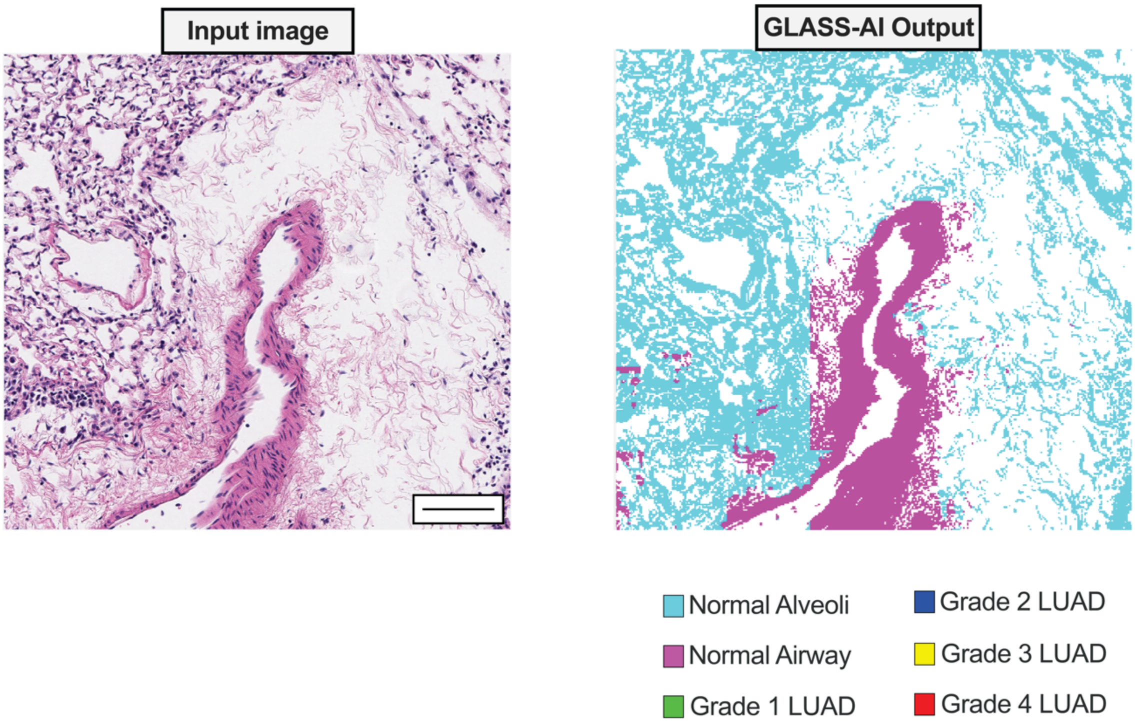

Supplementary Figure 3: Misclassification of smooth muscle cells surrounding blood vessels. An example image of a large blood vessel incorrectly labeled as “normal airway” (magenta) by GLASS-AI. GLASS-AI labeled a smaller blood vessel on the left of the image as “normal alveoli” (cyan). Blood vessels were not annotated explicitly in the training data set and therefore included in the “normal alveoli” masks generated by human raters. The scale bar represents 100 μm.

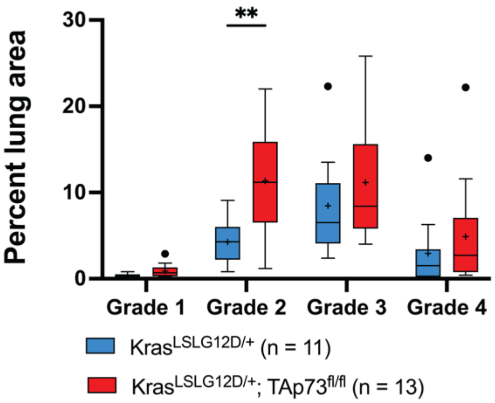

Supplementary Figure 4: Area of tumor grades in K and TK mice. The distribution of tumor burdens of individual K and TK mice was analyzed by Student’s t-test with Holm-Šídák correction after normalization to the total lung area in each slide. Boxplots are presented in Tukey style; a line at the median with IQR, crosses indicating the mean, whiskers showing the lesser of 1.5x IQR or most extreme values, and points indicating outliers > 1.5x IQR from the median. $*p <$ 0.05 for the indicated comparison between K and TK mice.

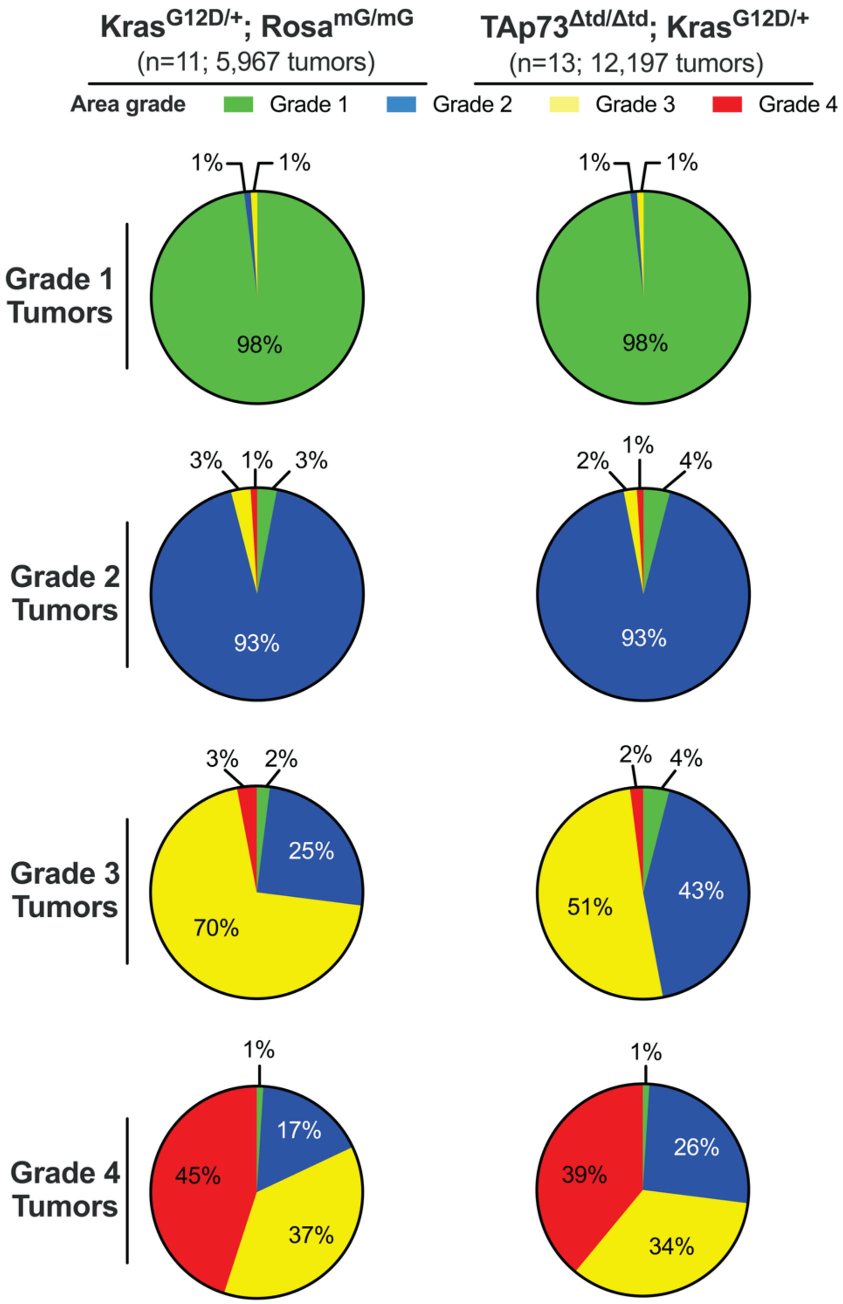

Supplementary Figure 5: Proportion of area grades within tumors of each grade in K and TK mice. Pie charts show the proportion of total area of each LUAD grade found within tumors of the indicated overall tumor grade. Overall tumor grades were assigned to individual tumors based on the highest tumor grade present that comprised ≥10% of the tumor's area.

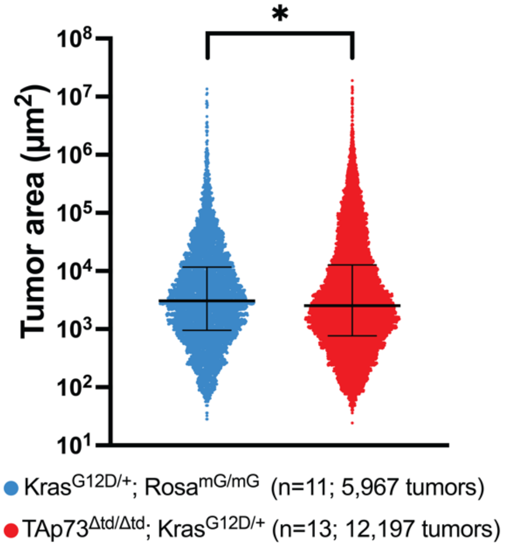

Supplementary Figure 6: Distribution of tumor sizes in K and TK mice. Analysis of individual tumor size distribution was performed by the Mann-Whitney U test due to high skewness and kurtosis of the data and significant differences in variance between groups even after log transformation. Lines represent median ± IQR. *$p < 0.05$ for the indicated comparison between K and TK mice.

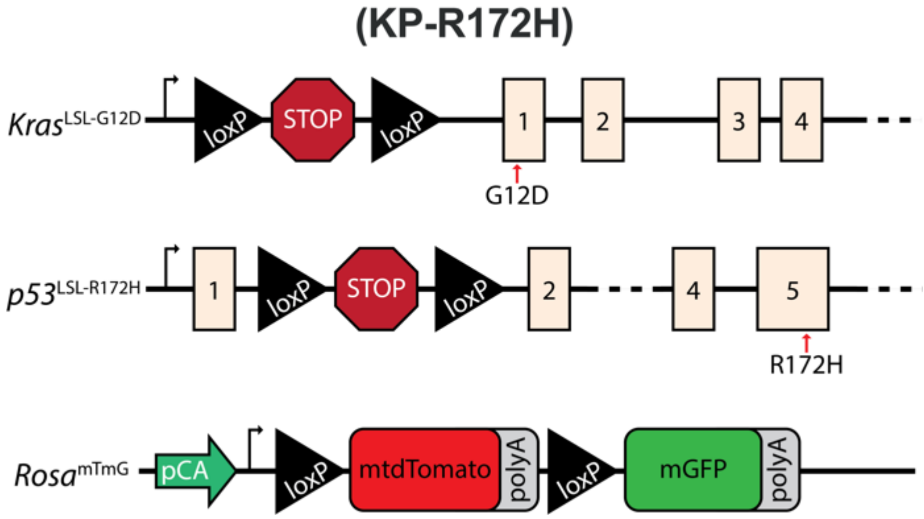

Supplementary Figure 7: Diagram of alleles in Kras$^{LSL\text{-}G12D/+}$; Trp53$^{LSL\text{-}R172H}$; Rosa$^{mTmG/mTmG}$ (KP-R172H) mice.

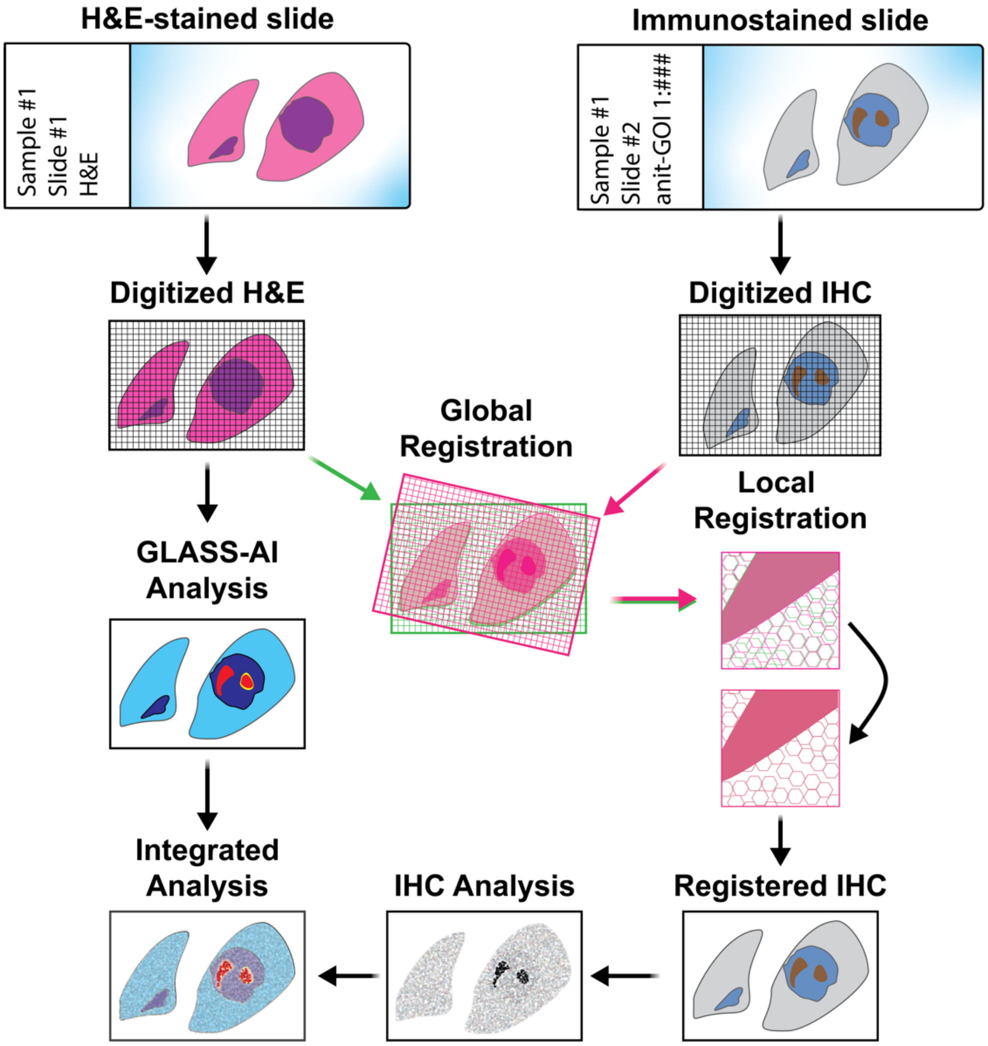

Supplementary Figure 8: Slide co-registration workflow.
