# Supplementary material for: Grading of lung adenocarcinomas with simultaneous segmentation by artificial intelligence (GLASS-AI)
Source: NPJ Precis Oncol. 2023 Jul 18;7:68. doi: 10.1038/s41698-023-00419-3 (PMC10354042; doi:10.1038/s41698-023-00419-3)
Supplement: Supplementary file 3 — REPORTING SUMMARY [file 41698_2023_419_MOESM3_ESM.pdf]

nature portfolio

Corresponding author(s): Elsa Flores (elsa.flores@moffitt.org)

Last updated by author(s): Mar 30, 2023

# Reporting Summary

Nature Portfolio wishes to improve the reproducibility of the work that we publish. This form provides structure for consistency and transparency in reporting. For further information on Nature Portfolio policies, see our Editorial Policies and the Editorial Policy Checklist.

## Statistics

For all statistical analyses, confirm that the following items are present in the figure legend, table legend, main text, or Methods section.

| n/a | Confirmed | |
|---|---|---|
| ☐ | ☒ | The exact sample size (*n*) for each experimental group/condition, given as a discrete number and unit of measurement |
| ☐ | ☒ | A statement on whether measurements were taken from distinct samples or whether the same sample was measured repeatedly |
| ☐ | ☒ | The statistical test(s) used AND whether they are one- or two-sided<br>*Only common tests should be described solely by name; describe more complex techniques in the Methods section.* |
| ☒ | ☐ | A description of all covariates tested |
| ☐ | ☒ | A description of any assumptions or corrections, such as tests of normality and adjustment for multiple comparisons |
| ☐ | ☒ | A full description of the statistical parameters including central tendency (e.g. means) or other basic estimates (e.g. regression coefficient) AND variation (e.g. standard deviation) or associated estimates of uncertainty (e.g. confidence intervals) |
| ☐ | ☒ | For null hypothesis testing, the test statistic (e.g. *F*, *t*, *r*) with confidence intervals, effect sizes, degrees of freedom and *P* value noted<br>*Give P values as exact values whenever suitable.* |
| ☒ | ☐ | For Bayesian analysis, information on the choice of priors and Markov chain Monte Carlo settings |
| ☒ | ☐ | For hierarchical and complex designs, identification of the appropriate level for tests and full reporting of outcomes |
| ☐ | ☒ | Estimates of effect sizes (e.g. Cohen's *d*, Pearson's *r*), indicating how they were calculated |

*Our web collection on statistics for biologists contains articles on many of the points above.*

## Software and code

Policy information about availability of computer code

| | |
|---|---|
| Data collection | Whole slide images were annotated using Leica ImageScope software v12.4.3.5008. Immunohistochemical staining was quantified using Definiens Tissue Studio software v2.7. |
| Data analysis | Grading of whole slide images was performed using the GLASS-AI software described in this report. A standalone version of the software for Mac and Windows is available at https://github.com/jlockhar/GLASS-AI along with the source MATLAB code. |

For manuscripts utilizing custom algorithms or software that are central to the research but not yet described in published literature, software must be made available to editors and reviewers. We strongly encourage code deposition in a community repository (e.g. GitHub). See the Nature Portfolio guidelines for submitting code & software for further information.

## Data

Policy information about availability of data

All manuscripts must include a data availability statement. This statement should provide the following information, where applicable:

- Accession codes, unique identifiers, or web links for publicly available datasets
- A description of any restrictions on data availability
- For clinical datasets or third party data, please ensure that the statement adheres to our policy

The datasets generated during and/or analyzed in this study are available from the corresponding author upon reasonable request.

## Human research participants

Policy information about studies involving human research participants and Sex and Gender in Research.

| | |
|---|---|
| Reporting on sex and gender | *Use the terms sex (biological attribute) and gender (shaped by social and cultural circumstances) carefully in order to avoid confusing both terms. Indicate if findings apply to only one sex or gender; describe whether sex and gender were considered in study design whether sex and/or gender was determined based on self-reporting or assigned and methods used. Provide in the source data disaggregated sex and gender data where this information has been collected, and consent has been obtained for sharing of individual-level data; provide overall numbers in this Reporting Summary. Please state if this information has not been collected. Report sex- and gender-based analyses where performed, justify reasons for lack of sex- and gender-based analysis.* |
| Population characteristics | *Describe the covariate-relevant population characteristics of the human research participants (e.g. age, genotypic information, past and current diagnosis and treatment categories). If you filled out the behavioural & social sciences study design questions and have nothing to add here, write "See above."* |
| Recruitment | *Describe how participants were recruited. Outline any potential self-selection bias or other biases that may be present and how these are likely to impact results.* |
| Ethics oversight | *Identify the organization(s) that approved the study protocol.* |

Note that full information on the approval of the study protocol must also be provided in the manuscript.

# Field-specific reporting

Please select the one below that is the best fit for your research. If you are not sure, read the appropriate sections before making your selection.

☒ Life sciences ☐ Behavioural & social sciences ☐ Ecological, evolutionary & environmental sciences

For a reference copy of the document with all sections, see nature.com/documents/nr-reporting-summary-flat.pdf

# Life sciences study design

All studies must disclose on these points even when the disclosure is negative.

| | |
|---|---|
| Sample size | Sample size for training data generation was determined by availability of animals and human raters.<br>Sample sizes for manual characterization was determined by availability of animals/slides and human raters.<br>Sample sizes for characterization by GLASS-AI were determined by availability of animals/slides. |
| Data exclusions | No data were excluded from our analyses. |
| Replication | Animals used for lung adenocarcinoma model characterization were drawn from multiple cohorts. |
| Randomization | No experimental randomization was necessary to complete this study. Groups were based solely on genotypes (mouse models) and existing clinical characteristics (human tissue microarray) |
| Blinding | Human raters were blinded to the genotypes of the mice during tumor grading for both training dataset generation and manual characterization of the mouse models. |

# Reporting for specific materials, systems and methods

We require information from authors about some types of materials, experimental systems and methods used in many studies. Here, indicate whether each material, system or method listed is relevant to your study. If you are not sure if a list item applies to your research, read the appropriate section before selecting a response.

## Materials & experimental systems

| n/a | Involved in the study | |
|---|---|---|
| ☐ | ☒ | Antibodies |
| ☒ | ☐ | Eukaryotic cell lines |
| ☒ | ☐ | Palaeontology and archaeology |
| ☐ | ☒ | Animals and other organisms |
| ☒ | ☐ | Clinical data |
| ☒ | ☐ | Dual use research of concern |

## Methods

| n/a | Involved in the study | |
|---|---|---|
| ☒ | ☐ | ChIP-seq |
| ☒ | ☐ | Flow cytometry |
| ☒ | ☐ | MRI-based neuroimaging |

# Antibodies

| | |
|---|---|
| Antibodies used | p-Mek1/2 (Ser221) (Cell Signaling Technology Cat# 2338, RRID:AB_490903; lot #9; 1:200)<br>p-Mapk (Erk1/2) (Thr202/Tyr204) (Cell Signaling Technology Cat# 4370, RRID:AB_2315112; lot #17; 1:400) |
| Validation | All antibodies were validated for immunohistochemistry on mouse tissue by the manufacturer. |

# Animals and other research organisms

Policy information about studies involving animals; ARRIVE guidelines recommended for reporting animal research, and Sex and Gender in Research

| | |
|---|---|
| Laboratory animals | All mouse models used in this study were generated on a C57BL6 background. Both male and female mice were included. Tumor formation was induced in animals between 8 - 10 weeks of age. Animals were collected 30 weeks after tumor induction. |
| Wild animals | The study did not involve wild animals. |
| Reporting on sex | Mice of both sexes were included in the model training data set and in the model characterization data set. However, sex was not analyzed as a covariate in these analyses. Animal cohorts were comprised of available appropriately aged animals (8 - 10 weeks) at the time of tumor initiation regardless of the sex of the animal. |
| Field-collected samples | The study did not involve field-collected samples. |
| Ethics oversight | Animal use was approved by the Institutional Animal Care and Use Commmittee of the University of South Florida. |

Note that full information on the approval of the study protocol must also be provided in the manuscript.
